# Urban Pest or Aussie Hero? Changing Media Representations of the Australian White Ibis

**DOI:** 10.3390/ani14223251

**Published:** 2024-11-13

**Authors:** Rebecca Scollen

**Affiliations:** School of Creative Arts, University of Southern Queensland, Toowoomba, QLD 4350, Australia; rebecca.scollen@usq.edu.au; Tel.: +61-7-4631-2774

**Keywords:** Australian White Ibis, bin chicken, pest, victim, survivor, hero, newspaper, media representation, content analysis

## Abstract

The Australian White Ibis (*Threskiornis molucca*) is an Australian native bird species whose traditional habitat is inland wetlands. Environmental factors have seen the species steadily relocate to the eastern coast of Australia over the last few decades, primarily settling in cities. In 2016, McKiernan and Instone identified that 70% of Australian newspaper reports about the urban ibises from 1998 to 2012 presented the birds as either pests or victims. Since then, the ibis populations have grown, leading to the ibis being voted one of the top ten most commonly seen urban backyard birds in 2019. Media representation both reflects and shapes public perception and understanding, so it is timely for a contemporary investigation into how the ibis is represented in Australian newspapers. Has newspaper representation of the Australian White Ibis changed since 2013? If so, what new narratives have emerged? A content analysis of 68 Australian newspaper items from 2013 to 2024 was conducted with results showing a decrease in pest and victim narratives and the introduction of two new positive narratives-survivor and hero. This finding evidences changes in media representation pointing to potential changes in public understanding of the ibis and the possibility of improved relations between humans and the native bird species.

## Simple Summary

Australian newspaper reporting (1998–2012) about the Australian White Ibis (*Threskiornis molucca*) typically presented the inland, native bird species as a pest, or as a victim, due to the birds’ relocation to urban environments along the eastern coast of Australia. Ibis populations have adapted well to their new habitat seeing a steady increase in bird colonies. With this increase has come complaints from human residents about the negative impacts of the birds in the shared environment. As media representation can both reflect and shape public perception and understanding, it is timely for a contemporary investigation into how the ibis is represented in Australian newspapers. Has newspaper representation of the Australian White Ibis changed since 2013? If so, what new narratives have emerged? A content analysis of 68 Australian newspaper items from 2013 to 2024 was conducted with results showing a decrease in pest and victim narratives and the introduction of two new positive narratives-survivor and hero. This finding demonstrates shifts in media representation pointing to the potential for change in public understanding of the ibis and the possibility of improved relations between humans and the native bird species.

## 1. Introduction

The ways media and popular culture re-present animals impact their lives as well as ours [1] (Merskin, 2024). According to media theorist, Stuart Hall [2] (p. 3), the way we make meaning of “people, objects and events” in the world is by encoding and decoding language. This constructionist approach to representation [2] (p. 11) argues that the language we use and the stories we tell shape our understanding of those around us. The media is a key institution for storytelling, with news reports informing public perception of ourselves, of others, and of actions and events. One species that has regular media mentions is the Australian White Ibis. This somewhat controversial Australian native bird species (*Threskiornis molucca*) traditionally lives in inland wetlands. However, environmental factors have seen the species steadily relocate to the eastern coast of Australia over the last few decades, primarily settling in cities. In 2016, McKiernan and Instone [3] identified that 70% of Australian newspaper reports about the urban ibises from 1998 to 2012 presented the birds as either pests or victims. Since then, the ibis populations have grown further leading to the ibis being voted one of the top ten most commonly seen urban backyard birds in 2019. Given the prevalence of the bird species in cityscapes and the subsequent increase in human interaction with the birds, it is timely to investigate how the Australian White Ibis is now represented in Australian newspapers. This investigation is prompted by the following inquiry: Has newspaper representation of the Australian White Ibis changed since 2013? If so, what new narratives have emerged? Framed by a constructionist approach to representation, this study employs content analysis of 68 newspaper items from 2013 to 2024 to then compare with the previous findings of the content analysis of 68 newspaper items from 1998 to 2012 undertaken by McKiernan and Instone [3]. This paper does not attempt to analyse the possible intentions of the newspaper journalists who have penned the reports, nor the possible reactions of the public to those reports. Such analysis is beyond the scope of this research project, which focuses on the content of Australian newspaper stories about the Australian White Ibis. The following provides further context for the Australian White Ibises’ relocation to cityscapes and the reported reception they received from humans already residing there.

### Australian White Ibis and the Move to the City

The Australian White Ibis (*T. molucca*) is an Australian native bird species protected by law. Growing up to 75 centimetres long [4] (p. 2) and “identifiable by their large white bodies, featherless black heads and long black downward-curved bill” [3] (p. 481), the Australian White Ibis (ibis) is an inland waterbird who naturally feeds on “saltwater and freshwater wetland invertebrates and fish” [5] (p. 290). Environmental factors such as drought and habitat loss due to “human encroachment” [3] (p. 482) have seen the ibis populations declining inland [6] (p. 1023) but increasing in cities along the eastern coast of the country as the birds relocate in search of food and water. Willis et al. [4] (p. 11) explain that “While urbanisation typically has negative impacts on biodiversity, many generalist bird species (including the ibis) can excel in an urban environment when provided the opportunity and resources.”

In fact, “birds seem ideally suited to an urban lifestyle because they can fly in and out of backyards and remnant bush … (in particular) the winners are the large-bodied birds” [7] (p. 231). The urban ibises “mostly occupy parks which support foraging, roosting and breeding … and landfills which are a major foraging resource” [8] (p. 1286). They have proven to be “highly adaptive to untraditional environments as they learn to live-with humans” [3] (p. 482) unthreatened by other animal predators. Not only have the ibises “habituated to humans, but (they) have learned to identify people as a source of food” [5] (p. 297). This sees ibises as regular visitors to cafes, picnic grounds, and streetscapes ready to pilfer leftovers with no fear of humans within close proximity.

The ibis drew little public attention until the early 2000s when the media began reporting their growing presence in urban landscapes. According to McKiernan and Instone [3] (p. 483), Australian newspaper coverage of the birds at this time was deemed ‘neutral’ in its attitude toward the birds. They noted that of the 68 newspaper items from 1998 to 2012, 20 (or 29.5% of the sample) were neutral or balanced in their reporting about the ibis. However, by 2003 onward the stories in the media had noticeably shifted to a pest narrative, with the birds no longer seen as “novel guests” but instead “native pests” [3] (p. 483). In fact, McKiernan and Instone [3] identified 35 out of the 68 newspaper articles (or 51.5% of the sample) from 1998 to 2012 presented the ibis as a pest.

“The pest category is a powerful narrative influencing modes of relating and demarcates positions of belonging. Urban pests are unwanted species positioned as out-of-place and whose presence is met with discursive and material reaction.”

[3] (p. 477).

Although an Australian native animal, many regarded the ibis as akin to an introduced species, and invasive and exotic pests [9] (p. 372). “Pest species are often perceived as a nuisance, negatively impacting the community, the economy, or environment” [4] (p. 1) as they move into new territory beyond their traditional geographical boundaries. Animals that are understood to be neither friend nor food, “are typically framed as living outside of the standard sphere of our actions and consideration … (and) do not always live in ways that easily align with human interests and human ideas as to the *proper* inhabitation of the Australian landscape” [10] (p. 154). When wild animals relocate to urban areas attempts are often made to expel them because they are considered to be “polluting, disruptive and discomforting” to humans [11] (p. 207). Yet, such movement is likely as human expansion and climate change impact the lives of flora and fauna and place an “increasing number of animals … into a liminal or contact zone” [12] (p. 226), blurring the boundaries of wild and domesticated, human and non-human. As Taffel and Holm [13] (p. xiv) state, “entanglements between humans and nonhumans looks set to be a central concern of the twenty-first century”.

As the ibis numbers continued to increase and their visibility in urban areas became more apparent, a “counter narrative of ibis as an environmental victim and refugee” emerged [3] (p. 483), with McKiernan and Instone [3] identifying 13 newspaper articles (or 19% of the sample) presented the ibis as a victim. Two-thirds of these articles were published in 2010 to 2012, indicating that the victim narrative appeared to be growing. By mid-2000 and onward for the rest of the decade drought ravaged the interior of the nation. This prompted some media outlets to call for compassion and understanding for the ibises as these inland waterbirds sought new homes with ready water sources, such as artificial wetlands [6] (p. 1024) available to them in cities. As the colonies of ibises grew in the urban settings, “sometimes numbering hundreds of nests and thousands of individuals” [4] (p. 2), so too did the number of complaints from human residents. Primary concerns raised pertained to their “noise and odour … (and their) stealing food from humans” [4] (p. 2), along with possible “disease transfer to humans, fauna displacement, and flora degradation” [3] (p. 482), as well as collisions with aircraft causing damage and injury [6] (p. 1024). However, by 2010 media reports began discussing possible ways to live with the ibises as victims of circumstance, rather than calling for the birds’ removal as had been primarily the focus in the past [3] (p. 484). Overall, McKiernan and Instone [3] (p. 483) identified that Australian print news media reporting about the ibis from 1998 to 2012 showed the ‘pest narrative’ outweighed the ‘victim narrative’ by almost 300%. They argued that “ibis presence in the city disrupts human positions of control as ibis challenge their placement and disrupt the conceptions of domesticated urban wilds” [3] (p. 476).

Historically a rare sight in urban settings [4] (p. 2), by 2019, the ibis was voted one of the top ten most commonly seen urban backyard birds [14]. “The ibis assumes such a highly visible urban presence that the species appears to be uncontained” [9] (p. 373). The ibises’ predilection for scavenging on human waste earned them a variety of unfortunate nicknames such as ‘tip turkey’, ‘dumpster chook’ and most commonly used, ‘bin chicken’ [9] (p. 373). Although referred to in such disparaging terms, the ibis was voted runner-up in the 2017 Australian Bird of the Year awards [15] (p. 47) and has “gained nation-wide attention and cultural notoriety” [9] (p. 374). The birds’ popularity is further evidenced by the “ever expanding spate of Ibis memorabilia, clothing and accessories, artefacts, murals, art and craft works, films, songs, tattoos and other cultural representations” [9] (p. 374). Given the increased awareness of the ibis and the arguably antagonistic or at times, jingoistic nature of our relationship with the species since the newspaper content analysis of McKiernan and Instone [3], it seems timely for an analysis of ibis representation in Australian newspapers from 2013 to 2024. Will the coverage continue to perpetrate a pest or victim narrative, or will it have changed concomitantly with Australians’ increased interaction with the ibis in the shared urban environment?

## 2. Materials and Methods

### 2.1. Theory of Representation and the Media

The focus on text and its meaning is an acknowledgement of the influential role discourse plays in communication and media. The language of discourse serves to legitimize ideology [16] (p. 466), perpetuating power relations [17] (p. 103) and shaping perception by making a position appear normal [18] (p. 8). Context and history inform discourse, and can change it over time, so to understand a particular discourse, context must be taken into account [19] (p. 277). Analysis of discourse can reveal the powerful application of language to frame issues in a manner which can “persuade, alter perception, or legitimise practices” [18] (p. 9). Language is never neutral; it constructs ”particular versions of reality, social identities and social relations” [17] (p. 96). Stuart Hall [20] (pp. xvii; xix) explains, “Language is the privileged medium in which we ‘make sense’ of things, in which meaning is produced and exchanged … We give things meaning by how we represent them-the words we use about them, the stories we tell about them, the images of them we produce, the emotions we associate with them, the ways we classify and conceptualize them, the values we place on them”.

The media, including newspaper reporting, “create, sustain and distribute mainstream ways of thinking” [21] (p. 16) through their use of language as representation. Language use makes sense of the world and “constructs rather than reflects ‘reality’” [22] (p. 192) generating narratives that “do not simply endorse existing values and forms of relation but … also open up novel ways of looking at things and new possibilities for action” [10] (p. 6). Mummery and Rodan [10] (p. 153) explain that narratives about animals and about our relations with them shape society’s “ideas and interactions with animals”. They assert that for narratives to be effective in building “identification, empathy, and the acceptance of the need for a change” they need to employ “emotional modes of appeal” or language that can sway and re-shape public perception [10] (p. 7). Thus, it is appropriate to investigate the word choices and narratives presented in the media to see how the ibis is represented as such language has the power to shape and to re-shape public views about the bird species.

Framed by a constructionist approach which understands that “we *construct* meaning using representational systems-concepts and signs” [2] (p. 11), or language, this paper utilises a quantitative and qualitative content analysis methodology to identify how many times the Australian White Ibis is referred to in Australian newspapers from 2013 to 2024, and how the bird species is represented as evidenced by the discourse of those newspaper items. By interrogating the language use through the process of coding, indications of how the media represents the birds and their relationship with humans in the urban landscape, emerge. Merskin [21] (p. 22) supports such an approach, as she states “Content and textual analyses of news coverage of human/wildlife interactions are useful in determining tone, perspective, and risk …”

### 2.2. Content Analysis

“Content Analysis is a structured and demonstrable approach to drawing meaning from media in context” [23] (p. 89). By engaging with texts as data (e.g., newspapers, speeches, archives, etc.), this methodology is premised on the understanding that “valuable information about particular phenomena” [24] (p. 128) can be revealed. Content analysis can be applied quantitatively and/or qualitatively with both approaches aiming to sort text “to identify similarities and differences, patterns, and associations, both on the surface and implied within” [24] (p. 128). The quantitative method determines the frequency of key categories within the text under review to determine their prevalence. This assists the researcher “to answer questions about *how many*” in an attempt to draw realistic conclusions of the text’s content [25] (p. 11). Qualitative content analysis then “delves into a deeper examination”, identifying concepts and constructs alluded to by the recurring key words or phrases which are utilised for substantiation [24] (p. 128).

In content analysis “themes, patterns, and codes should be identified and grouped in mainly two different ways: inductively or deductively” [26] (p. 90). This study incorporates a deductive approach as the data are “analysed in a top-down process using codes chosen earlier (not from the data itself) and supported by theory or previous research” [26] (p. 90). This analytical approach is commonly referred to as “*directed* content analysis” [23] (p. 86), which is structured in its examination and categorisation of content because it is an extension of “existing theory and the prior description of phenomena” [26] (p. 91). As a directed content analysis, informed by previous investigations but occurring now at a later stage, it is possible that the data under review does not neatly fit into the predetermined categories, with new relevant data highlighted. This can lead to additional categories to those already existing and directing the content analysis [26] (p. 91). The content analysis previously undertaken by McKiernan and Instone [3] identified three central ibis narratives present in the newspaper items: 1. pest, 2. victim, and 3. neutral/balanced (information sharing without a noticeable bias or perspective). This study applies these three codes to determine whether the narratives remain relevant and whether their inclusion has increased or decreased in newspapers since 2013. The McKiernan and Instone [3] study went on to include personal interviews at selected case study sites to further frame the narratives as detailed in the newspaper items they collated. This study does not undertake additional qualitative research of this kind, but instead focuses solely on a comparative analysis of the narratives in the two sets of newspaper items.

### 2.3. The Sample

Australian newspapers available via online databases, Factiva and Australian Newspapers Online, were sourced up until 31 August 2024 using search terms ‘Australian White Ibis’ and ‘bin chicken’. Establishing the search parameters to commence from 2013 enabled the data to be compared with that of McKiernan and Instone [3], who undertook a content analysis of 68 Australian newspaper articles to understand their representations of the Australian White Ibis from 1998 to 2012. Ninety-six newspaper articles published between 1 January 2013 and 31 August 2024 were retrieved. Any of these articles where the content was repeated verbatim in multiple newspapers were reduced to a count of one, and any articles that made passing reference to ibises but the news stories did not pertain specifically to ibises were removed. This second stage of filtration saw the number of articles reduced to 68 for this study’s analysis.

Of the 68 newspaper articles, fifteen (or 22%) were drawn from the UK-owned *Mail Online* [27,28,29,30,31,32,33,34,35,36,37,38,39,40,41]; eleven (or 16%) were drawn from News Corp’s *Courier Mail* [42,43,44,45,46,47,48,49,50,51,52]; five (or 7%) were from News Corp’s *Macarthur Chronicle* [53,54,55,56,57], and from *The Advertiser* (News Limited) [58,59,60,61,62]; four (or 6%) were from *The Guardian* (Guardian Media Groups) [63,64,65,66]; and from Nine’s *Sydney Morning Herald* [67,68,69,70] and from News Corp’s *Canterbury Bankstown Express* [71,72,73,74]; three (or 4.5%) were from News Corp’s *news.com.au* [75,76,77]; two (or 3%) were from the UK-owned *Daily Mail Australia* [78,79], and from News Corp’s *NCA Newswire* [80,81] and *The Australian* [82,83]; and one (or 1.5%) was from local independent newspapers *Illawarra Flame* [84] and *Sydney Sentinel* [85], from News Corp’s *Central* [86], *Daily Telegraph* [87], and *Fairfield Advance* [88], and *Townsville Bulletin* [89], from the British Broadcasting Commission’s *BBC News Sydney* [90], from the Australian Broadcasting Commission’s *ABC News* [91], from Time Out Media’s *Time Out* [92], and from Nine’s *Leeton Irrigator* [93]. The vast majority of the articles were produced by Sydney-based publications with a national reach, with the exception of News Corp’s *Courier Mail* in Brisbane (capital city of Queensland) and the *Townville Bulletin* in North Queensland (1350 km north of Brisbane), along with Nine’s *Leeton Irrigator* in the Riverina Region of New South Wales (550 km west of Sydney), and News Limited’s *Advertiser* based in Adelaide, the capital city of South Australia. This illustrates the sample includes chiefly national newspapers, along with a small number of state-based and regional publications.

## 3. Results

As explained in Materials and Methods, once the 96 newspaper articles were reduced to 68 unique items, the process of analysing the content of each began. Firstly, the number of times ‘Australian White Ibis’ and ‘bin chicken’ were used was recorded. Then, pre-determined codes of pest, victim, and neutral/balanced provided the lens to examine words used in each article and the overarching storylines presented. This approach identified key words and phrases that were emotive and as such expressed a particular viewpoint that could be associated with the pest or victim narrative. Typically, the pest narrative was present through the use of negative terms for the ibis and the reporting of negative impacts on humans. The victim narrative was illustrated through word choices that showed the ibis as negatively impacted by humans. In most cases, these news stories contained either the pest or the victim narrative. However, occasionally, items included a combination of these two narratives as indicated by the overlap of key terms and sentiments expressed. When two narratives were present in one news item, this was given the new category of ‘mixed narrative’. By using content analysis in this way for all 68 newspaper articles, it soon became apparent that two other additional codes were needed to illustrate the new categories of ibis narrative that were emerging. These new codes were ‘Ibis as survivor’ and ‘Ibis as hero’. The word choices indicating a survivor narrative spoke positively about the birds’ ability to adapt and thrive in a new environment. The hero narrative was reflected in key terms celebrating the ibis and its positive impact on human culture. Again, there were the occasional newspaper articles that contained each of these two narratives or contained one of these two narratives in combination with the pest or victim narrative. These were all tallied under the code of mixed narrative. Finally, there were a small number of articles that were identified as neutral/balanced as their word choices were to provide facts and were lacking in emotive connotation.

**Figure 1:**
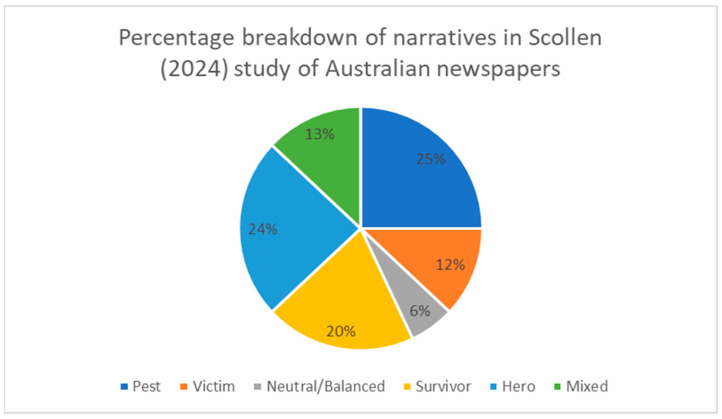
Percentage distribution of narratives in Scollen’s study about the ibis in Australian newspapers from 2013 to 2024.

Table 1 below provides a quick summary of the tally of narratives present in the newspaper articles from 2013 to 2024. The tally is then presented in graphical form (Figure 1) to indicate the percentage breakdown of narratives within the study.

### 3.1. Pest

By comparing the data of the two content analyses, it is apparent that the occurrence of the pest narrative has significantly reduced since the McKiernan and Instone [3] study. Their research saw 35 articles present the ibis as a pest, whereas this research identified half that amount with 17 articles (or 25% of the total sample) from 2013 to 2024. Of this number, the vast majority of the articles were published from 2013 to 2019, with only three news items since that time. This demonstrates that not only has the pest narrative diminished since the earlier research was undertaken, but that it has almost disappeared within the last five years.

Content analysis revealed key words and phrases within the 17 newspaper articles that not only presented the ibis in a negative light but also demonstrated antagonism toward the bird species. Some chief examples of ‘pest’ words, and the number of times they are mentioned across the sample, are as follows:

- Problem (10);
- Stinky/smelly (8);
- Bin chicken (7);
- Infestation (6), health risk (6), make a mess (6), need to be managed/management (6);
- Urban pest (5), noisy (5);
- Destroying/destruction (4), disease/salmonella (4);
- Invasion/invade (3), complaints (3), overpopulation/too many (3);
- Annoyance/nuisance (2), tip turkey (2), disgusting (2), birds we love to hate/most hated bird (2), pesky (2), harass humans (2);
- Taken over (1), dirty (1), widespread (1), unbearable (1), dumpster diver (1), flying rat (1), scavengers (1), horrible creatures (1), don’t belong (1), aggressive (1), gross (1), swarm (1), havoc (1), terrorised (1).

Words such as these provide an indication of the disdain for the bird species. The storylines concern residents’ complaints about the increasing number of ibises in the human urban/suburban environment who reside in colonies, scavenge from human rubbish, and make a mess. The people featured do not like the birds and do not want them living in the same environment as they do. By way of illustration, some direct quotes from the newspaper articles are included here: “… forced to endure around-the-clock screeching, pungent smells and tonnes of bird droppings” [50]; “They are destroying the trees down at the Eagle Vale pond. We need to get rid of them” [53]; and “A preschool in Sydney’s south is being terrorised by a population of Ibis birds who are destroying the property” [80].

The first actual reference to ‘pest’ or ‘urban pest’ is made in two articles in 2015 [54,60] and is then used in some articles following. The greatest number of articles with a pest narrative was also published in 2015, with most of these providing regular updates about the problem of birds in a particular location (Lake Gillawarna in Bankstown NSW). The articles not only refer to the residents’ complaints but to the efforts of the local government to ‘manage’ the ibises, via culling, oiling eggs, and destroying abandoned nests. This same location features in the McKiernan and Instone [3] study, so the references to Bankstown and its unhappy relationship with ibises are a continuation some years on from the articles they analysed. For example, as quoted in the *Canterbury Bankstown Express* in 2015 and again in 2016, “… many of the ‘horrible creatures’ had since ditched the man-made lake for life on the streets” [72] and “They’re useless, they come in the backyard and make a mess, especially when washing’s outside … They’re everywhere–I think they’re taking over” [73].

The year 2016 sees the first reference to ‘bin chicken’ [73], the now-common nickname for the ibis. This derogatory term for the bird species refers to the ibises’ scavenging behaviour observed at rubbish tips (or waste facilities) and in bins located on the street and in public parks. The first references to claims that ibises create ‘health risks’ to the community and can be ‘aggressive toward humans’ occurred in 2017. For example, in ‘Ibis colony poses health risk to Hyperdome neighbours’ [50], it is stated that “A Queensland Health spokesman warned residents to stay away from the site saying the birds could transmit diseases including salmonellosis. Ibis may also harass humans and occasionally become aggressive towards humans for food.” Prior to this time, the chief concerns about the ibises were smell, noise, and mess. It is possible the idea of disease and aggression is prompted by the growing number of ibises in various urban locations and the evident lack of fear the birds have for humans. By 2018, the first reference to ibises as birds that ‘we love to hate’ is registered [75]. This phraseology suggests that hatred toward ibises is common and is an emotion that we share as an urban population.

### 3.2. Victim

By comparing the data of the two content analyses, it is apparent that the occurrence of the victim narrative has also significantly reduced since the McKiernan and Instone [3] study. Their research saw 13 articles present the ibis as a victim, whereas this research identified just over half that amount with eight articles (or 12% of the total sample) from 2013 to 2024. Of this number, the vast majority of the articles were published in 2023, with only two news items prior to that time. The prevalence of the victim narrative in this one year is the result of four articles published progressively as reports about an individual’s attack on an ibis and his arrest, trial, and conviction are revealed. If not for the violent actions of this one man and the updated reports of his punishment, the victim narrative in 2023 and for the entire decade under review, would be almost non-existent. In *Mail Online* [35], [the man] “… allegedly stuffed the bin chicken into his backpack and then rode to his unit in Sydney’s eastern suburbs where he decapitated it and hung it up in his shower on Tuesday, in preparation to eat the native bird … charged with animal cruelty and harming or attempting to harm a protected animal … Members of the public attempted to stop the birdnapping … Witnesses told police the bird appeared to have an injured beak and was bleeding … The white Australian ibis, colloquially referred to as a bin chicken, is a protected species … it cannot be harmed or interfered with”.

Content analysis revealed key words and phrases within the eight newspaper articles that positioned the ibis as a victim of direct human attack. Some chief examples of ‘victim’ words, and the number of times they are mentioned across the sample, are as follows:

- Deaths/killed (8);
- Protected species (6), animal cruelty (6);
- Distressed (3), harm (3);
- Injuring/injury (2), rescue (2), concerns (2), attack (2), torture (2), offence (2);
- Sickening (1), faced challenges (1), worried (1).

Words such as these provide an indication there is sympathy for the birds and their plight. The storylines condemn those who deliberately injure or harm ibises, and they repeat that the birds are protected under Australian law.

### 3.3. Neutral/Balanced

By comparing the data of the two content analyses, it is apparent that the occurrence of the neutral/balanced narrative has significantly reduced since the McKiernan and Instone [3] study. Their research saw 20 articles discuss the ibis in a neutral or balanced manner, whereas this research only identified four articles (6% of the total sample) from 2013 to 2024. This appears to demonstrate that general information sharing for the purposes of educating the public about the bird species is no longer perceived as necessary by newspaper outlets. This is likely because ibises since the beginning of this century have become an ever-familiar sight in urban environments and public awareness of the bird species is widespread, in part due to the neutral/balanced narratives throughout the 2000s. Of the four articles in this study that were identified as presenting information about ibises in a neutral manner, their storylines consisted of how the movement of the ibis can give indications of how the T Rex dinosaur moved [65]; observation of an ibis with unusual colouring [31]; calls for community members to report ibis sightings for census data [59]; and the misunderstanding of the ‘bin chicken’ term, as used in a popular Australian children’s television program, by some overseas viewers [78].

### 3.4. Survivor

Regular occurrence of key words and phrases, along with overarching storylines that neither fitted the pest nor victim narrative, led to the identification of a new category of ‘ibis as survivor’. The survivor narrative emerged in 2017 in the article ‘Ibis are here to stay says Australian Museum researcher’ [86] and continued to be employed in a number of articles through to 2022. Fourteen newspaper articles (or 20% of the total sample) were identified as presenting the ibis as a survivor, which was only three short of matching the number of ibis as pest articles within the study period. Some chief examples of ‘survivor’ words or phrases, and the number of times they are mentioned across the sample, are as follows:

- Adapt to urban environment (8);
- Thriving/population increase (4), survivors/survival (4);
- Evolution (2), superior scavenger (2);
- Here to stay (1), remarkable (1), transformed (1), call the city home (1), modified their habitat (1), resilient (1), iconic yet despised (1), symbol of fighting against the city (1), triumph against the odds (1), adapt-or-die (1), respect (1) imbecile humans (1), integrate (1), your city, our bins (1), reborn (1), do not fear humans (1), safer (1).

Words such as these provide an indication of respect and empathy for the bird species and a developing sense of identification with the ibis. The storylines praise the ibises for their resilience and for their successful adaptation to the human urban/suburban environment, which has seen their populations survive and thrive. There is acknowledgement that they have been forced to scavenge in cityscapes, but rather than discussing this in victim terms, they are applauded for their survival skills and their ability to draw upon new-found resources to feed and to nest.

Some examples of direct quotes from newspaper articles are as follows:

“The bin chicken is the symbol of fighting against the city and trying to triumph against the odds, so we have a very soft spot for them. They’re survivors–adapt-or-die embodied in one filthy, flying cockroach … The ibis has found an alternative habitat basically in the middle of the city and bin juice is their new estuary fishing ground … The bin chicken is not that dissimilar to your average Sydney punter trying to get through the day despite the myriad horrific things being done to us”

[70].

“I started thinking about what the daily life would be like for an ibis and how it compares to our lives in terms of finding food and housing … There is a struggle within the story of the ibis … They have had to flee their highlands, and have been forcibly displaced, and it’s a story about what it’s like to integrate …”

[69].

“One of the big revelations we’ve had over the last few years is discovering just how sophisticated they are, they’re treating cities as yet another habitat alongside things like forests and wetlands … The coronavirus pandemic has proven that Australia’s ‘bin chickens’ can survive without relying on rubbish left behind by humans … When the pandemic reduced foot traffic across the nation’s cities, the birds were forced to adapt to the changing environment”

[29].

### 3.5. Hero

Regular occurrence of key words and phrases, along with overarching storylines, that neither fitted the pest, victim, nor survivor narrative, led to the identification of another new category of ‘ibis as hero’. In this context, heroes are understood to be courageous and bold and revered for their many virtues [94] (pp. 50–51). They are spoken of positively and are celebrated for their actions and achievements. The hero narrative first emerged in 2017, when one report [34] called the public to action to vote for the ibis to win the annual Bird of the Year award and another later report [28] stated the ibis was runner-up in the annual competition. The hero narrative continued to be employed in a number of articles through to 2024. Sixteen newspaper articles (or 24% of total sample) were identified as presenting the ibis as a hero, which is one percentage off matching the pest narrative and which outweighs the other published narratives within the study period. Some chief examples of ‘hero’ words or phrases, and the number of times they are mentioned across the sample, are as follows:

- Proposed mascot for the 2032 Brisbane Olympic Games (3);
- Iconic (2), winner (2), favourite (2), celebrate (2), appreciate (2), beautiful (2), famed/famous (2);
- Glorious bin chicken (1), culture and identity (1), important (1), really cool (1), special ability (1), majestic heron (1), gorgeous (1), ingenious method of eating cane toads (1), clever birds (1), unfair reputation (1), grateful for wonderful job (1), Australia’s fabulous flamingo (1), love (1), emblem (1).

Words such as these provide an indication that there is a growing, positive attitude toward the bird species and some actually celebrate the ibis. For example, in ‘Bin chicken crowned Brisbane’s icon in Reddit art prize’ [47], “Ibises have become a much more appreciated local icon in recent years with people embracing our ‘bin chickens”. Storylines have moved beyond a discussion of how the birds have successfully survived in their new habitat, and now highlight their influence on popular culture and Australian urban identity. The ibis now features in public artworks [67], television programming [70], and fiction [83] as well as merchandise [27]. The ibis was awarded runner-up in the 2017 Australian Bird of the Year awards [28] and is proposed as the mascot for the upcoming 2032 Olympic Games to be held in Brisbane [61]. The ibis as a species is further endorsed for successfully killing and eating cane toads (*Rhinella marina*); a poisonous, introduced species which has become an invasive pest in Australia [90]. It is within some of the newspaper articles that present a hero narrative where the ibises are described as beautiful, majestic, and gorgeous [79], whereas the more common past narrative of pest saw the birds described as dirty, smelly, and horrid [71]. The birds’ appearance and behaviours have remained the same. It is the newspaper representations of the ibis that have altered. A couple of direct quotes below provide further examples of how the ibis is positively represented and referred to in the following:

“I think a lot of people in Australia would see bin chickens all the time and they become pests so if you show someone a picture of an ibis, once you find a way to beautifully illustrate and enhance those unique features, they take on a different perspective and they start to appreciate the animal”

[68].

“In a move that has reignited Queensland’s affection for the ibis, or ‘bin chicken’, a Brisbane artist’s recent rooftop stunt has bolstered a push to make it the official mascot for the 2032 Olympic and Paralympic Games. Ryan Forster gained national attention by placing an ibis sculpture holding a XXXX can on the Castlemaine Perkins Brewery on 14 January. This earned praise from brewery director Shane McIntyre, who gave the sculpture a permanent position on the roof. Brisbane Lord Mayor Adrian Schrinner has become a proponent of the bin chicken’s Olympic bid, proposing a bin chicken statue for City Hall”

[45].

### 3.6. Mixed Narrative

**Figure 2:**
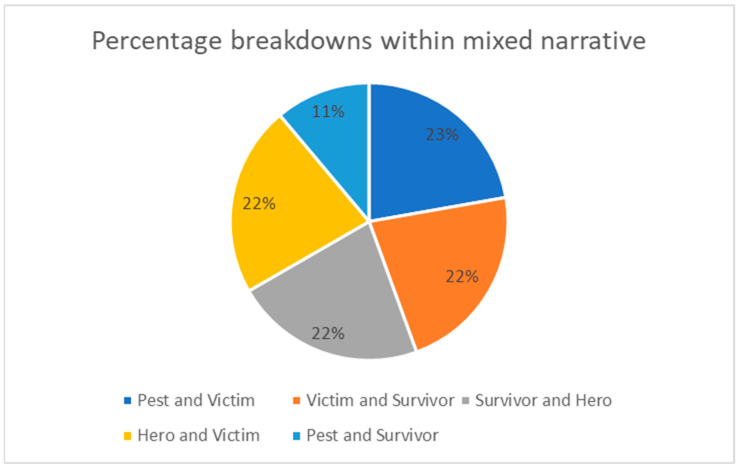
Percentage distribution of mixed narratives in Scollen’s study about the ibis in Australian newspapers from 2013 to 2024.

Of the 68 newspaper articles, nine (or 13% of the total sample) were identified as including a mixed narrative. This means they contained words and messages that presented more than one narrative (either pest, victim, survivor, or hero). Figure 2 (below) illustrates the distribution of mixed narratives within the nine newspaper articles. There were only two narratives present in any one article. The only combination of narratives not identified was pest and hero. Perhaps as these categories are positioned at each end of the narrative spectrum, a combination of the two in one article would seem incongruous with the story told.

Within the three articles including the pest narrative, words are used such as the following:

- Overpopulation (3), management (3), bin chicken (3);
- Complaints (2), noise (2), smelly/odour (2);
- Infestation (1), dirty (1), tip turkey (1), trash vulture (1), winged foe (1), scabby (1), intrusive (1), horrid (1), evil looking beak (1).

Such terms position the ibis as a pest due to the large number of them, their reliance on human waste for food, and their odour, noise and appearance perceived as unacceptable. However, within the mixed narrative, this perception of pest is tempered with an acknowledgement of their victim status, which sees them living in unnatural habitats due to changed environmental conditions beyond their control. An example of language use forming the pest and victim mixed narrative in one of the newspaper articles now follows:

“The Australian White Ibis is a big, dirty white critter with a hooked and evil-looking beak … They are loathed … I had a half-arsed theory that birds were all heading to the CBD to eat leftover delicious organic doughnuts and gozlemes in the wake of festival season. Not so. We’ve changed the landscape of the state, the country, in myriad of ways that affect how our native wildlife live, eat, breed and move. In short, all of our human behaviours have come home to roost”

[62].

The victim narrative is identified in six articles by the use of words or phrases, such as the following:

- Population decrease in natural habitat (3);
- Environmental degradation (2), human behaviour changed the landscape (2);
- Negative impact (1), become dependent on artificial food sources (1), misunderstood (1), entangled in line (1), forced to exist on the streets (1), animal cruelty (1), protected species (1), harm (1), killed (1), distressed (1).

Such terms position the ibis as a victim due to human-induced degradation of their natural habitat, and the harms caused to them by residing in the urban environment, including unnatural food sources and instances of cruelty. However, within the mixed narrative, this perception of the innocent victim is sullied by the pest narrative (two articles), which points to the negative impacts the birds have in the urban environment they have habituated. The victim narrative was also shown to be combined with the new category of ‘survivor’ in two of the newspaper articles. In these instances, the perception of an innocent victim is bolstered by an acknowledgement of their survivor status, which sees them successfully adapting and thriving in the urban environment despite the many challenges. Finally, a combination of victim and hero narratives (two articles) report that whilst the birds have positive traits, they are negatively impacted by human-induced changes. All six articles provide a public reminder that the ibises are only living in urban environments because of negative human impacts on their natural habitats.

An example of word usage forming the victim and survivor mixed narrative in one of the newspaper articles now follows:

“The toes were so constricted against the leg that it could only hobble on the resulting stump. Such is the fate of the urban ibis: driven from its home range in the Murray-Darling by a lack of water … forced to eke out an existence on the streets of our cities … Yet the ibis persists … The more I see of these city ibis the more I admire their resilience and adaptability”

[14].

The survivor narrative is identified in five articles by the use of words or phrases, such as the following:

- Survivor/survive (6);
- Adaptability (3);
- Thriving/increasing (2), Aussie battler (2);
- Evolution (1), commonly seen (1), resilience (1), persistence (1), no fear of humans (1), can-do attitude (1), transformation (1).

Such terms position the ibis as a survivor due to the ability to adapt to a new environment and to evolve and thrive in the new setting. However, within the mixed narrative, this perception of survivor is either: begrudgingly acknowledged when combined with the pest narrative which points out their negative impacts on humans (one article); strengthened when combined with the victim narrative to remind the reader of the ibises struggles (two articles); or lifted to the level of celebration when combined with the hero narrative as their positive characteristics are highlighted (two articles). An example of language forming the survivor and pest mixed narrative, and an example of word usage of the survivor and hero mixed narrative now follow:

“Although it’s sometimes used affectionately, “bin chicken” still seems a bit of an unfair nickname given that ibis are a native bird species that are simply making the best of a colonised, urbanised environment … Bin Chicken Island really is the epitome of the ibis’s can-do attitude … There has been a rapid expansion of ibis population between 2018–2020 … Despite the birds being native, there are some concerns the population may be getting a smidgen out of hand, with water around the island looking poor with algae growth–which could be related to the bird’s droppings. The community has also raised concerns about the noise, smell and behaviour of the colony …”

[92].

“The sudden and spectacular transformation of the Australian White Ibis from a shy and obscure species of remote inland wetlands, to a creature capable of not only surviving the city but owning it, has been one of nature’s greatest mysteries. …its secret weapon is (now) clear: it has lost the natural fear that has stopped a suite of similar wild birds from successfully making the jump to big city life …”

[85].

Finally, within the articles that have a mixed narrative, the hero narrative is identified four times by the use of words or phrases, such as the following:

- Iconic (1), graceful (1), decorative (1), devoted monogamous partners (1), great gardeners (1), love ibises (1), fight locust plagues (1), true romantics (1), amazing (1), live happily with ibises (1), urban success story (1), cult-hero (1), elegant (1), better at city life than we are (1), bloody classic (1), really smart (1).

Such terms position the ibis as a hero due to the benefits they bring to the environment, along with their attractive physical appearance, and their success in city living. Within the mixed narrative, this perception of the hero draws upon either the survivor (two articles) or victim (two articles) narratives to provide a context for the celebrated birds who have triumphed in the face of significant environmental change and overcome other negative human impacts. An example of language forming the hero and victim mixed narrative now follows:

“It might seem like ibis numbers are increasing in our cities. In their natural, rural habitats, however, ibises are facing big declines … “Tip turkeys”, “sandwich snatchers” or “feathered rats”–the poor old Australian White Ibis gets a bad rap … The next time you see a white ibis strolling across a lawn, be thankful … They are not only helping with the gardening, they are also fighting plagues and looking after their families … There are reasons to love the birds”

[42].

### 3.7. Australian White Ibis Versus Bin Chicken

The formal name, ‘Australian White Ibis’, is referred to 74 times in the newspaper items analysed in this study. It appears from 2013 through to 2024, with the most mentions (22) in the year 2015. As the years go by the number of times the birds are referred to as Australian White Ibises reduces to eight mentions in 2017, 2020 and 2021, and then further reduces to six mentions in 2022, five mentions in 2023, and one mention so far in 2024. The steady reduction in the use of ‘Australian White Ibis’ aligns with the growing use of the name ‘bin chicken’ in newspaper articles.

In 2016, the term ‘bin chicken’ was included in a newspaper article for the first time, with 132 instances of this phrase recurring over the following years. The term originated as a derogatory nickname for the Australian White Ibis and featured regularly in the newspaper items which presented an ‘ibis as pest’ narrative. The nickname connoted ibises as dirty, thieving, unhygienic, and messy. However, just as the discourse of the newspaper articles shifted over time to the more positive ‘survivor’ and ‘hero’ narratives, so too did the colloquial meaning of ‘bin chicken’ in these news items. The raiding of bins for food is understood as a necessity for the birds as survivors in a new habitat, so their behaviour remains problematic but is also perceived as acceptable. This has led to a shift in the connotation of ‘bin chicken’ with many viewing it as an affectionate term for the ibis, aligning it with Australians’ fondness for the resilient ‘Aussie battler’ [9] (p. 372) or survivor. The ibis as ‘bin chicken’ has now also achieved a cult-hero status within Australian popular culture [9] (p. 374) evidenced by a plethora of merchandise and artwork that specifically refer to the ibis as a ‘bin chicken’. The ‘bin chicken’ is mentioned in celebratory terms in the newspaper items that present the ‘ibis as hero’ narrative, for example as “a bloody classic” [66]. Here the bird is perceived as a “symbol of the city” [85] and as a living representation of the Australian sense of humour, which is typically self-deprecating and anti-authoritarian [95] (p. 92).

## 4. Conclusions

This research sought to address the following questions: Has newspaper representation of the Australian White Ibis changed since 2013? If so, what new narratives have emerged? A comparison of the 68 newspaper items from 1998 to 2012 with the 68 newspaper items from 2013 to 2024 shows a dramatic decrease in the ‘ibis as pest’ and ‘ibis as victim’ narratives in media reporting. It also sees a large drop in the number of newspaper articles within the category of neutral or balanced narrative of the ibis. The void left by these significant declines has been filled with two new narratives—‘ibis as survivor’ and ‘ibis as hero’. Together, these two positive narratives make up almost half (44%) of the total sample of newspaper items since 2013. This demonstrates a noticeable and widespread shift in discourse in the articles, potentially reflecting a change in public sentiment for the bird species. In the last five years alone, the trend away from the earlier narratives to the new more positive representations of the ibis is evident. The number of times the various narratives have been published since 2020 are as follows: pest (3); victim (6); survivor (8); hero (11); mixed (3); and neutral (2). As indicated earlier, the number of victim references since 2020 is somewhat inflated by ongoing coverage of one particular incident. Of the three mixed narratives, these combinations consisted of the following: survivor and hero (1); pest and survivor (1); and hero and victim (1). Again, even with this very small sample the survivor and hero narratives dominate over the last five years. Therefore, in recent times, there has been a marked difference in the way the ibis has been represented by Australian newspapers spanning locations throughout New South Wales, Queensland, and South Australia.

The formal name of Australian White Ibis is referred to regularly in the news items but steadily decreased in mentions from 2016 onward. Instead, the slang term ‘bin chicken’ takes preference when referring to the bird species. At first, it is employed pejoratively for the ibis but over time is used as an affectionate and celebratory reference to the bird in keeping with the change to survivor and hero narratives. The ‘bin chicken’ has become an icon of the Australian urban landscape, whose successful scavenging points to the problem of escalating human waste and other negative impacts we have on the natural environment. As such, the term semiotically points to the ibis but also to the negative impact we have had on this native bird.

The changing ibis narratives in Australian newspapers since 1998 point to changing public perception of the birds and our relationships with them in a shared urban environment. After a brief period (1998–2002) of non-judgemental reporting of ibis populations arriving in urban areas, the dominant discourse of news articles to follow (2003–2019) presented the ibis as an unwelcome pest and openly expressed disdain for the birds. One can speculate this was due to the increasing visibility of the ibises living in what was considered human environments. The birds’ growing presence and subsequent impact in urban areas was likely unsettling to residents, as this change disrupted the ‘normal’ way of life and challenged “humans positions of control … (and) conceptions of domesticated urban wilds” [3] (p. 476). Those communities positioned by rivers and other waterways appeared to be most appealing to the birds as they relocated, and thus these communities (such as in the Canterbury Bankstown catchment) feature most strongly in the news items.

The items with the ‘ibis as victim’ narrative, although fewer in number, counteracted this negativity by being sympathetic to the birds’ plight because they had been forced to leave their natural habitat. This narrative appeared mostly from 2010 to 2012, likely as a result of a decade’s worth of ravaging drought making the ibises’ inland homes unhabitable. The tussle between pest and victim/disdain and sympathy appears to have more-or-less ended by the introduction of the ‘ibis as survivor’ narrative (2017–2022). This discourse reflects a growing respect for the birds as they successfully adapt to a new environment, modifying their habitat, food, and behaviours to thrive in urban areas. Building on this respect, came the ‘ibis as hero’ narrative first indicated in 2017 and then steadily featuring in newspaper articles through to 2024. This narrative sees humans celebrating the ibis and beginning to identify with the bird as a symbol of the city and as an Australian icon. The year 2017 is a transition year where pest and victim narratives were reduced and survivor and hero narratives emerged within newspaper articles. This timing coincides with the ibis being voted runner-up in the Bird of the Year Awards, with the rise of ibis memorabilia in popular culture, and with the inclusion of ibis as a subject within art works, films, and songs [9] (p. 374). The growing positive representation in the newspaper articles is in keeping with the positive sentiments reflected in these cultural artefacts, and by the public’s consumption of them. Also, by this point in time, the bird species will have been co-habiting with humans for at least two decades, and so the positive media representation may provide an indication of the public’s increased tolerance of the birds within the shared urban environment.

Content analysis has illustrated that the ways in which the ibis has been portrayed in newspaper reporting have dramatically changed over time. Whilst this native bird species have not altered in appearance nor behaviour since moving to the city, the media representation of the ibis has seen it transform from an urban pest to an Aussie hero within the last decade. Although now celebrated as a hero, the ibis’ cult-hero status remains chiefly predicated on the public’s embracing of the perceived negative (pest) attributes of the bird. Scavenging from bins and making a mess, snatching food from unsuspecting humans, undeterred and unfearful, sporting dirty feathers and unpleasant odour, these characteristics of the ibis in the city are recognised as gross but also as embodied, empowering symbols of self-deprecation, anti-authoritarianism, and rascal-like can-do attitude. Australian culture takes pride in the underdog, the low in status, and the battler. We might mock and laugh at the unsophisticated and the vulgar, but this national sense of humour also conveys affection and comradery for the subject of our joke [9] (p. 377). We celebrate those who triumph against the odds and do so on their own terms. Thus, at present, the heroic representation of the ibis is primarily centred within the ironic. The ibis is ultimately an anti-hero at this time because although not represented with “outstanding or exceptional qualities, the anti-hero still possesses good qualities that set an example, so he manages to win … admiration” [94] (p. 51).

The shift in narratives within newspaper reporting of the ibis has implications for the native bird species and for humans co-habiting within the urban environment. As indicated by Mummery and Rodan [10] (pp. 6–7), narratives can “open up novel ways of looking at things and new possibilities for action” and can build “identification, empathy, and the acceptance of the need for a change”. This paper has evidenced the significant reduction in the pest and victim narratives representing the ibis over the last decade; instead, they have been replaced with more positive narratives aligned with survival and celebration. As representation [2] is argued to shape the way we understand the world around us, then the public perception of the ibis in the Australian urban landscape appears to be changing to a more favourable perspective concomitant with the changing narratives featured in the newspaper articles. The potential for humans to be more accepting of the birds, to recognise their status as urban residents, and to begin to positively identify with them opens up possibilities for a more respectful, shared existence between the two animal species in the future.

### Recommendations

As identified early on in this paper, this research has its limitations due to its sole focus on the collation and analysis of Australian newspaper representations of the ibis within a specified period of time. Content analysis reports on numbers and trends. The paper has not sought to detail the worldview of the newspapers nor of their journalists. It has not sought to undertake a reception study to ascertain the views of individuals and communities within the urban Australian environment. Nor has it sought to directly enter the debates concerning animal agency, animal ethics, and multispecies entanglements. It is recommended that future research builds from the findings of this particular study, to provide further qualitative data to engage with contemporary debates. There is also the capacity to draw upon the plethora of merchandise and creative artworks that feature the ibis to gain an understanding of the narratives at play (beyond the media) that may reflect or shape our understanding of this native bird species.

Finally, it is recommended that content analysis of media representation of the ibis be employed in another ten years to monitor the number of media mentions and to identify what narratives are present. If media reporting has reduced or perhaps ceased after a time, this may provide an indication that the birds’ presence in the city is well accepted and thus considered non-newsworthy. If media reporting has continued over the following decade, will there be a change in narrative about the ibis? Will the pest and victim narratives have ceased altogether? Will the survivor and hero narratives still feature? Perhaps new narratives will have emerged? Undertaking a longitudinal study employing the same methodology generates insight into how our understanding of this native bird species has altered and grown.

## Figures and Tables

**Table 1:** Overview of ibis representation from 2013 to 2024. Note the additional categories of Ibis as ‘survivor’, Ibis as ‘hero’, and mixed, which were not present in the McKiernan and Instone [3] study.

Narrative	2013–2014	15	16	17	18	19	20	21	22	23	24	Total
Ibis as ‘pest’	1	5	2	1	3	2			1	2		17
Ibis as ‘victim’		2								6		8
Ibis as ‘survivor’				2	2	2	3	3	2			14
Ibis as ‘hero’				2	1	2	1	2	4	2	2	16
Mixed		3		1	1	1	1	1		1		9
Neutral/balanced	1				1			1		1		4
Total	2	10	2	6	9	7	5	7	7	12	2	68

## Data Availability

The data presented in this study are available in article.
